# Profiles of HIV Risk, Sexual Power, and Decision-Making among Sexual Minority Men of Color Who Engage in Transactional Sex: A Latent Profile Analysis

**DOI:** 10.3390/ijerph18094961

**Published:** 2021-05-07

**Authors:** S. Raquel Ramos, David T. Lardier, Donte T. Boyd, José I. Gutierrez, Eliana Carasso, David Houng, Trace Kershaw

**Affiliations:** 1 Rory Meyers College of Nursing, New York University, New York, NY 10016, USA; erc424@nyu.edu (E.C.); dh2630@nyu.edu (D.H.); 2 Department of Individual, Family, and Community Studies, College of Education and Human Sciences, University of New Mexico, Albuquerque, NM 87131, USA; dlardier@unm.edu; 3 Department of Psychiatry and Behavioral Sciences, University of New Mexico School of Medicine, University of New Mexico, Albuquerque, NM 87131, USA; 4 College of Social Work, The Ohio State University, Columbus, OH 43210, USA; boyd.465@osu.edu; 5 National Clinician Scholar, Philip R. Lee Institute for Health Policy Studies, University of California San Francisco, San Francisco, CA 94118, USA; jose.gutierrez2@ucsf.edu; 6 School of Public Health, Yale University, New Haven, CT 06520, USA; trace.kershaw@yale.edu; 7 Center for Interdisciplinary Research on AIDS, Yale University, New Haven, CT 06520, USA

**Keywords:** transactional sex, HIV, pre-exposure prophylaxis, MSM, sexual relationship power, young adult, latent profile analysis, sexual behavior

## Abstract

Though the transmission of HIV is preventable, there were still 37,968 new documented cases in the United States in 2018. HIV incidence is disproportionate in sexual minority men of color. The purpose of this study was to examine sexual relationship power risk profiles to identify distinct subgroups within the profiles who carry the highest HIV risk. Latent class profile analysis was used to identify subgroups of sexual minority men of color at the highest risk for contracting HIV based on their sexual power profiles. Among 322 sexual minority men, we identified four latent profiles. Profile 1: Low transactional sex and high power (*n* = 133; 14.3%); Profile 2: Transactional sex, high decision-making in sexual relationships, and low control in sexual relationship (*n* = 99; 30.7%); Profile 3: Low transactional sex, low decision-making, and moderate control (*n* = 43; 13.4%); Profile 4: High transactional sex and low power (*n* = 47; 14.6%). LPA was useful to identify distinct subgroups based on measures of sexual risk and relationship sexual power. Findings carry significant implications for developing tailored strategies to increase HIV knowledge and related HIV prevention and risk reduction services for sexual minority men of color who engage in transactional sex.

## 1. Introduction

Though the transmission of HIV is preventable, there were still 37,968 new documented cases in the United States (US) reported in 2018 [1]. The highest incidence of HIV is in sexual minority men (SMM) of color (of color being those who identify as either ethnic and/or racial minorities) [1]. The US National Institutes of Health’s definition of sexual minorities includes individuals who identify as lesbian, gay, bisexual, transgender, intersex, asexual or those with same-sex or same-gender attractions or behaviors who may not self-identify with the aforementioned identities [2]. In 2018, The Centers for Disease Control (CDC) reported that 69% of all US reported HIV incidence occurred in SMM [1]. According to a 2018 report, the CDC also found that African Americans accounted for 42% of new HIV diagnoses while making up only 13% of the US population; the Latinx population accounted for 27% of new US HIV diagnoses, but only constituted 18% of the US population [1]. HIV continues to disproportionately affect ethnic/racial SMM. One factor that can heighten HIV risk, within this population, is sexual relationship power.

Sexual relationship power is the perceived decision-making ability that an individual has concerning the power and control they have within a sexual relationship [3]. Sexual power is linked to an individual’s sexual self-efficacy; so increased power, in turn, is linked to the increased likelihood of safer sex behaviors [4]. Conversely, decreased relationship power has been correlated with higher HIV risk [5]. One factor contributing to decreased relationship power is transactional sex. Transactional sex, also referred to as exchange sex, “i.e., selling sex for money, drugs, a place to stay, or other survival needs” [6], places an individual at risk for low relationship power as it creates a power imbalance. This imbalance can discourage engagement in safe sex practices [5,6] and may increase engagement in substance use during sex, which exponentially increases the risk of HIV transmission [6,7].

Correspondingly, in the literature, high sexual relationship power indicates a greater ability to communicate sexual preferences, especially in safe sex practices such as condom use and substance use abstinence [3,8,9,10,11]. For example, in a study examining sexual power in Black women, findings suggested that Black women had a greater ability to communicate sexual preferences and negotiate condom use when sexual relationship power was high [12]. One potential contributor to increased sexual relationship power is higher HIV knowledge. HIV knowledge refers to the personal knowledge an individual has about the ways HIV is transmitted between persons [13]. Increased HIV knowledge can empower sexual self-efficacy and, in turn, HIV preventive practices [14,15]. In many studies, HIV knowledge is a significant predictor of HIV preventive practices and is linked to a decrease in overall HIV transmission risk, as increased knowledge may help guide the use of safe sex practices [10,15,16,17].

If high sexual relationship power can enable safer sexual practices [15], then decreased power may be a catalyst for increased HIV risk. For example, sexual relationship power is negatively impacted in the context of transactional sex. This is because “safer sex must be negotiated alongside fantasies, motivations, power, and desire” of the client who may incentivize the transactional sex worker to act against their self-interests [6,18]. This type of dynamic, for example, was implied in a study where homeless youth who engaged in transactional sex were more likely to engage in riskier sex behaviors, such as condom-less sex [19].

Clear linkages have been made between decreased relationship power and increased instances of condom-less sex and substance use, numerous sexual partners, and participation in transactional sex [6,9,10]. The trauma of previous relationships often has enduring effects throughout an individual’s life. Consequently, SMM who have endured forms of abuse were more likely to engage in risky sexual behaviors such as substance use [20], condom-less sex [21], and transactional sex [6]. Substance use can exacerbate low sexual relationship power since substance use can affect an individual’s decision-making ability and also limit one’s agency for sexual negotiation [22]. If substance dependency develops, the financial burden of sustaining dependency may increase financial need to engage in transactional sex [23]. Low sexual relationship power engenders a cycle of decreased agency and greater HIV risk-taking.

In addition to the risks associated with low sexual relationship power, HIV risk is compounded in SMM of color for several reasons. For example, stigmas surrounding HIV and identifying as SMM have contributed to underutilization of HIV testing in communities with high HIV risk [24,25]. Inconsistent or delayed testing in SMM of color has been documented because of decreased access to health care [18,24,26]. Decreased HIV testing in SMM of color, specifically those who engage in transactional sex, may increase HIV transmission rates in persons who have not been tested and are unsure of their HIV serostatus [6,24].

Although there is literature discussing the relationship between sexual relationship power and HIV risk, existing literature on sexual relationship power, transactional sex, and HIV transmission has primarily addressed women [4,8,9,10,12,15,27]. Consequently, few studies have examined the interconnectedness between HIV knowledge, sexual relationship power, transactional sex, and safe-sex behaviors in SMM of color. The purpose of this study was to examine the risk profiles of sexual minority men of color using latent profile analysis and to identify the distinct subgroups within the profiles who carry the highest HIV risk.

## 2. Materials and Methods

This study used cross-sectional, secondary data of 322 SMM of color ages 18–34 who resided in the United States. The primary study, which was conducted from 2017 to 2019, was a multi-step, mixed methods study. The purpose of the primary study, entitled HIV Oral Testing Infographic Experiment or HOTIE, was to design and then test the usefulness, ease of use, and comprehension of an HIV self-testing infographic. A national recruitment panel service was used to recruit a US-based sample of self-identified SMM of color. Details of the primary study are reported on clinicaltrials.gov (NCT04061915). As part of this analysis, we were interested in examining the risk profiles that affect HIV risk in young, ethnic and racial, sexual minority men. The study was approved by the Institutional Review Board at New York University.

### 2.1. Measures

#### 2.1.1. Latent Profile Variables

**Transactional sex.** Transactional sex was measured using a single-item question (During the last 3 months, did you have oral or anal sex with a man that gave you money, drugs, other goods (e.g., good, clothing, transportation), or a place to stay for sex?) measured dichotomously (*Yes* = 1, *No* = 0). Approximately, 20% of the sample identified exchanging sex for money, drugs, or other goods.

**Sexual relationship power.** The Sexual Relationship Power Scale is a 23-item measure that examines the participants’ perceived ability to have decision-making power and control in their sexual relationship (sample items: Who usually has more say about whose friends to go out with? My partner tells me who I can spend time with.) [10,28]. Responses are collected using a Likert-type scale from strongly disagree (1) to strongly agree (4). Due to concerns with the internal reliability of the measure during preliminary analyses, factor analysis was conducted on the measure to support the validity of the current sample group. Factor analysis using varimax rotation indicated that the two-dimension factor structure accounted for 70% of the variance, with an Eigenvalue of 7.85. Factor loadings ranged from 0.62 to 0.80. Three questions were removed due to poor performance in the factor structure with loadings <0.40 and suggested an increase in alpha-level reliability. The remaining item responses were summed to yield a total score of *Power in Sexual Relationships: Control* ranging from 12 to 48 (M = 36.79 ± 8.85; Cronbach’s alpha = 0.89) and a total score of *Power in Sexual Relationships: Decision-Making* ranging from 7 to 21 (M = 14.02 ± 2.46; Cronbach’s alpha = 0.88). For latent profile analyses, continuous responses were collapsed into quartiles based on original Likert-type response categories, which is consistent with prior research on latent profile analyses [29]. The quartile ordinal responses for Control (r = 0.92, *p* < 0.001) and Decision-Making (r = 0.90, *p* < 0.001) were highly correlated with the original continuous total scores, indicating collinearity.

#### 2.1.2. Predictors of Latent Class Profile Groups

**HIV knowledge**. The Brief HIV Knowledge Questionnaire (HIV-KQ18) [13] is an 18-item *true* or *false* response measure that distinguishes understanding about HIV transmission, prevention, and consequences (sample items: Coughing and sneezing DO NOT spread HIV; A person will NOT get HIV if she or he is taking antibiotics.). Correct responses were coded as “1” with incorrect responses coded as “0”. Prior studies have demonstrated good internal consistency ranging from 0.75 to 0.89 [13]. The measure has been identified as suitable for those with low health literacy [13]. For the current study, responses to the 18 items were summed to yield a total score on HIV knowledge ranging from 0 to 18.00 (M = 11.51 ± 4.76, Cronbach’s α = 0.84).

**Access to HIV testing outcomes.** Access to HIV testing outcomes was measured using a single item question: “I consider myself to be?” Responses were collected using five ordinal response options HIV test was negative (1), HIV test was positive (2), HIV test was inconclusive (3), HIV test results have not been received (4) and have not had an HIV test (5). Approximately 85% of the sample identified having a negative HIV test and 14.9% did not have their HIV testing results.

**Number of sexual partners in the past 6 months.** Number of sexual partners in the past 6 months was measured using a single-item continuous response question: “Fill in the number of persons who you have had sex with over the last 6 months.” Participant responses ranged from zero (0) to over 40 sexual partners (M = 4.48 ± 10.77).

**Safe sex behavior.** The Safe Sex Behavior Questionnaire is a 27-item measure that examines the frequency of use of recommended practices that reduce one’s risk of exposure to and transmission of HIV (sample items: I ask potential sexual partners about their sexual histories. If I know an encounter may lead to sexual intercourse, I carry a condom with me.) [30]. Responses were collected using a four-point Likert-type scale from *Never* (1) to *Always* (4). For the purposes of this study, five-item responses for *Safe Sex Behavior: No condom use* (M = 12.85 ± 3.99; range = 5 to 20.00; Cronbach’s alpha = 0.82) and four-items responses for *Safe Sex Behavior: Drug use during sex* (M = 2.33 ± 1.44; range = 1.00 to 8.00; Cronbach’s alpha = 0.80) were each combined to yield total scores, with higher values representing less condom use and more drug use during sex, respectively.

#### 2.1.3. Covariates

Several *sociodemographic covariates* were tested as statistical controls. Covariates included latent profile analysis and multinomial logistic regression models and were retained based on performance in the model (see [31]). These covariates included age (in years), race–ethnicity, education completed, employment status, and individual income. *Age* was measured in years. *Race–ethnicity* was categorized dichotomously (*Yes* = 1, *No* = 0) using several questions that asked participants their race–ethnicity including Hispanic/Latinx, Black/African American, American Indian, Asian, and Middle Eastern. *Education* completed was characterized using seven items categorized as *less than high school* (1), *high school graduate/GED* (2), *some college* (3), *2-year degree* (4), *4-year degree* (5), *professional degree* (e.g., Physician), (6); *doctorate* (e.g., PhD). *Individual income* was categorized using 7 items ranging from *less than $10,000 USD per year* (1) to *more than* $*150,000 USD per year* (7).

## 3. Analysis

Following the examination of descriptive statistics and bivariate correlations, latent class profile analysis (LPA) was used to identify distinct subgroups based on measures of relationship sexual power including exchange for sex and sexual relationship decision-making power and sexual relationship control. After completing LPA modeling, heterogeneity was examined among profile groups between sociodemographic variables and indicators of sexual health including participating in HIV testing, HIV knowledge, number of sexual partners in the past 6-months, condom use during sex and no drug use during sex. LPA identifies unobserved subgroupings based on specific indicators. LPA modeling were conducted using Latent Gold 5.1 [29]. LPA is a person-entered analysis that is methodologically stronger and allows the researcher to categorize and uncover participation within subgroups and generate outcomes that align theory and how groups of individuals function relative to others within the same population [32]. Latent profile measurement indices (e.g., transactional sex and sexual relationship decision-making power and sexual relationship control) were transformed into quintiles so that data were standardized and maintained ordinal response scales for the purpose of latent class modeling Six cluster groups were modeled in latent profile analyses model and model fit was assessed for each of the latent class cluster models to determine the most parsimonious and best fitting cluster model to the sample data, as well as the model that captured the largest amount of total association between observed indicators. The likelihood ratio chi-squared statistics (L^2^), Bayesian Information Criterion (BIC), Akaike Information Criterion (AIC), number of parameters, and classification error were used as comparative indicators to assess model fit [29].

Between group tests were conducted next on sociodemographic characteristics among profile groups, followed by mean-level between group analyses of indicators of sexual health (e.g., transactional sex, HIV knowledge, HIV testing outcomes availability, number of sexual partners in the past 6-months, sexual risk including no condom use during sex and drug use during sex) among profile groups. Last, multinomial logistic regression analyses were conducted between indicators of sexual health on profile group membership. This model controlled for sociodemographic characteristic covariates. Covariates with *p*-values of ≤0.20 were included in the multinomial logistic regression [33] and retained based on performance in these models [31]. Traditional levels such as 0.05 can fail in identifying variables known to be important [33]. These sets of analyses were conducted in Stata v. 15 software.

## 4. Results

### 4.1. Sociodemographic Characteristics and Bivariate Correlation Matrix

Table 1 shows all of the participants identified as sexual minority men. Participants’ ages ranged between 18 and 34 years (M = 26.35 ± 4.66), with most participants between 25 and 34 years of age (65.0%). Participants were predominantly Hispanic/Latinx (49.0%) and African American (37.9%). For those individuals who identified as having “White” race, they have *also* identified as being of Latinx ethnicity or having more than one racial/ethnic identity that is not exclusively of European descent. Participant education ranged from less than a high school education (2.0%) to doctoral degree (1.2%), with 38 percent having obtained a four-year college degree (29.5%) or professional degree (8.8%). A larger proportion of participants were employed full-time (52.4%) and 57% had an income of USD 10,000 to USD 39,999, with 18% having an income of less than USD 10,000 per year and 3.4% having an income of more than USD 150,000 per year. All participants had an HIV test in the 6 months prior to participating in the survey with 85.1 percent having a confirmed negative HIV test result.

Table 2 presents bivariate correlation matrix among main analytic variables. Results indicate that power in sexual relationships: control was positively correlated with power in sexual relationships: decision-making (r = 0.15, *p* < 0.01), access to HIV testing outcomes (r = 0.10, *p* < 0.05), and HIV knowledge (r = 0.17, *p* < 0.05) and negatively correlated with transactional sex (r = −0.19, *p* < 0.01), sexual risk: no condom use during sex (r = −0.17, *p* < 0.01), and sexual risk: drug use during sex (r = −0.35, *p* < 0.01). Power in sexual relationships: decision-making was negatively correlated with transactional sex (r = −0.15, *p* < 0.05) and sexual risk: no condom use during sex (r = −0.17, *p* < 0.01), and positively correlated with access to HIV testing (r = 0.10, *p* < 0.05). Transactional sex was negatively correlated with HIV knowledge (r = −0.10, *p* < 0.05) and positively correlated with number of sexual partners in the past 6-months (r = 0.21, *p* < 0.01) and sexual risk: drug use during sex (r = 0.27, *p* < 0.01). Access to HIV testing outcomes was positively correlated with number of sexual partners in the past 6 months (r = 0.12, *p* < 0.01) and sexual risk: drug use during sex (r = 0.15, *p* < 0.01). HIV knowledge was negatively correlated with sexual risk: drug use during sex (r = −0.11, *p* < 0.05). Finally, sexual risk: no condom use during sex was positively correlated with sexual risk: drug use during sex (r = 0.22, *p* < 0.01).

#### 4.1.1. Latent Profile Analysis: Sexual Risk and Sexual Power Profiles

**Figure 1:**
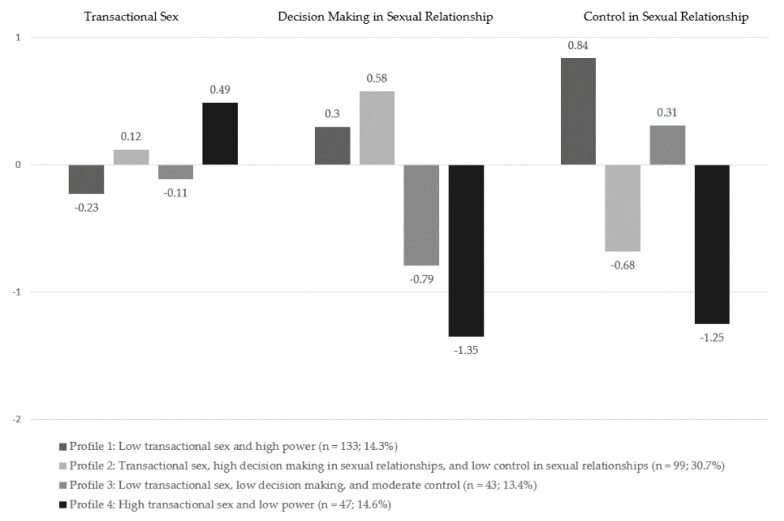
Sexual power profiles using standardized means.

Interpreter indicators of model fit (Table 3) were examined and showed that the four-cluster model provided the optimal model-to-data fit to the sample data. Bootstrapping was then conducted to obtain model fit estimates [34]. Fit statistics for the best-fitting four-cluster model were as follows: *L*^2^ = 10.32, BIC = 2006.93, AIC = 1935.21, and the bootstrap *L*^2^ *p*-value *=* 0.59 (see Table 3). The four-cluster model provided a standard *R*^2^ value of 0.78, indicating that these four latent class clusters account for a large proportion of the variance in these three indicators. Individual cases were assigned to latent class clusters using standard-modal classification (20). Only complete cases were assigned to classes (*n =* 322). The four identified profiles included: Profile 1: *Low transactional sex and high power* (*n* = 133; 14.3%); Profile 2: *Transactional sex, high decision-making in sexual relationships, and low control in sexual relationships (n* = 99; 30.7%); Profile 3: *Low transactional sex, low decision-making, and moderate control* (n = 43; 13.4%); Profile 4: *High transactional sex and low power (n* = 47; 14.6%). A visual representation of profiles using standardized measures of the study variables is presented in Figure 1.

Profile 1 was identified as *Low transactional sex and high power* due to low transactional sex (M = 0.05 ± 0.22), and high mean responses on control in sexual relationships (M = 44.32 ± 3.33) and decision-making in sexual relationships (M = 14.75 ± 1.46). Profile 2 was identified as *Transactional sex, high decision-making in sexual relationships, and low control in sexual relationships,* due to identification in engaging in transactional sex (M = 0.17 ± 0.37), though lower than Profile 4: *High transactional sex and low power*. Participants also identified high decision-making capacities during sexual relationships (M = 15.49 ± 2.17) and low mean rates of control during sexual relationships (M = 30.72 ± 6.68). Profile 3 was identified as *Low transactional sex, low decision-making, and moderate control* due to low mean responses of engaging in transactional sex (M = 0.09 ± 0.29), low decision-making in sexual relationship (M = 12.04 ± 1.04), and moderate mean rates of control in sexual relationships (M = 39.62 ± 4.39). Lastly, Profile 4 was identified as *High transactional sex and low power* due to highest mean rates of exchange for sex (M = 0.30 ± 0.46) and lowest rates of decision-making (M = 10.68 ± 2.03) and control in sexual relationships (M = 25.72 ± 5.16) when compared to other profile groups.

#### 4.1.2. Between-Group Differences on Sociodemographic Characteristics among Profile Groups and Mean-Level Differences of Profile Groups among Indicators of Sexual Health

Next, we tested profile group differences among sample sociodemographic characteristics (see Table 1). Statistically significant differences were noted on employment status (*χ*^2^ = 25.78, *p* = 0.01) and HIV testing (*χ*^2^ = 4.24, *p* = 0.05). Table 4 presents the mean-level group differences between latent profile groups on indicators of sexual health including HIV knowledge, number of sexual partners in the past 6 months, safe sex behavior: no condom use during sex, and safe sex behavior: drug use during sex, condom use, and drug use during sex. Statistically significant differences were present on HIV knowledge (F [4] = 1.12, *p* = 0.05) with those in Profile 1: *Low transactional sex and high power*, displaying greater mean rates of HIV knowledge compared to other profile groups (*M* = 13.44 ± 4.39), and those in Profile 4: *High transactional sex and low power* having the lowest mean rates of HIV knowledge compared to other profile groups (*M* = 10.90 ± 5.06). Statistically significant differences were present on number of sexual partners (F [4] = 2.05, *p* = 0.04), with those in Profile 1: *Low transactional sex and high power* displaying lowest mean rates of number of sexual partners compared to other profile groups (*M* = 2.37 ± 7.75), and those in Profile 4: *High transactional sex and low power* having the highest mean rates of number of sexual partners compared to other profile groups (*M* = 17.42 ± 8.01). Similar group differences were also noted on safe sex behavior: no condom use during sex (F [4] = 2.44, *p* = 0.04) and safe sex behavior: drug use during sex (F [4] = 15.96, *p* < 0.001). Those in Profile 1: *Low transactional sex and high power* displayed high mean rate of safe sex behavior: no condom use during sex (*M* = 13.49 ± 4.47), and those in *Profile 4: High transactional sex and low power* had higher mean rates of safe sex behavior: drug use during sex (*M* = 3.34 ± 1.70) when compared to other profile groups.

#### 4.1.3. Multinomial Logistic Regression Analysis

Lastly, we examined the association between indicators of sexual health including HIV knowledge, HIV testing outcome availability, number of sexual partners in past 6 months, safe sex behavior: no condom use during sex, and safe sex behavior: drug use during sex, on profile group membership using multinomial logistic regression analyses (see Table 5). Sociodemographic factors were included as covariates in the analysis. Profile 2: *Transactional sex, high decision-making in sexual relationships, and low control in sexual relationships* was identified as the reference category. Results indicate that HIV testing was positively associated with membership in Profile 1: *Low transactional sex and high power* (aOR = 2.57, 95% CI = 1.88, 3.01), indicating that for everyone one-unit increase in HIV testing, there was a 157% greater odds of membership in Profile 1: *Low transactional sex and high power* and Profile 3: *Low transactional sex, low decision-making, and moderate control* (aOR = 0.77, 95% CI = 0.22–0.99) and Profile 4: *High transactional sex and low power* (aOR = 0.38, 95% CI = 0.13, 0.87). HIV knowledge was positively associated with membership in Profile 1 (aOR = 1.11, 95% CI = 1.08, 1.93) and Profile 3 (aOR = 1.04, 95% CI = 1.01, 1.15), and a negative association with membership in Profile 4 (aOR = 0.93, 95% CI = 0.92, 0.99). The number of sexual partners in the past 6 months was positively and significantly associated with membership in Profile 4 (aOR = 1.52, 95% CI = 1.32, 3.20), indicating that for every one-unit increase in the number of sexual partners in past 6 months, there was a 52% greater odds of membership in Profile 4. The number of sexual partners in past 6 months was negatively associated with membership in Profile 1 (aOR = 0.78, 95% CI = 0.31, 0.99) and Profile 3 (aOR = 0.49, 95% CI = 0.08, 0.77). Safe sex behavior: no condom use during sex was positively associated with membership in Profile 4 (aOR = 1.12, 95% CI = 1.09, 1.24) and negatively associated with membership in Profile 1 (aOR = 0.88, 95% CI = 0.80, 0.98). Safe sex behavior: drug use during sex was also positively associated with membership in Profile 4 (aOR = 1.92, 95% CI = 1.46, 2.51) and negatively associated with membership in Profile 1 (aOR = 0.52, 95% CI = 0.39, 0.68) and Profile 3 (aOR = 0.64, 95% CI = 0.33, 0.72).

## 5. Discussion

The purpose of this study was to examine the latent profiles of 322 emerging adult SMM of color ages 18 to 34 in the United States and to identify profiles that were categorized as having the highest risk. Profile 1 participants were identified as having high rates of sexual relationship power and low transactional sex engagement. Profile 2 participants were identified as engaging in transactional sex and having high relationship decision-making, but low control. Profile 3 participants had low rates of transactional sex but had low relationship decision-making and moderate control. Profile 4 had high rates of transactional sex and low overall sexual relationship power. Our risk profile findings suggest that engagement in transactional sex and having low control (as seen in Profiles 2 and 4) were risk factors for unsafe sex practices and, transitively, HIV transmission. Findings also suggested that high relationship power, high decision-making, and low transactional sex (as seen in Profile 1) was associated with low HIV risk.

### 5.1. Transactional Sex

Our findings suggested that participants from Profiles 2 and 4, who reported having transactional sex and lack of relationship control, were more likely to engage in unsafe sex behaviors. Similarly, participants in Profile 3, those who also reported low decision-making power but did not engage in transactional sex, were also more likely to engage in unsafe sex behaviors. Consistent with the literature, condom-less sex was the most common risk behavior in Profiles 3 and 4, for those who reported having little decision power regarding sexual behaviors [8]. In this study, transactional sex in Profiles 2 and 4 was associated with unsafe sex behaviors, such as a high number of sexual partners and condom-less sex. This has been linked to increased rates of HIV transmission [35].

In a cross-sectional survey of 20 US cities, HIV transmission and transactional sex in SMM were examined [35]. In total, 13.2% of participants who had engaged in transactional sex in the past 12 months, tested positive for HIV but were unaware of their HIV serostatus [35]. However, 5.6% of participants in this study who did not engage in transactional sex were HIV-positive, but unaware of their serostatus [35]. Additionally, 60.7% of all participants who engaged in transactional sex were unsure about the HIV status of their last male sexual partner [35]. Conversely, only 37.5% of participants who did not engage in transactional sex reported not knowing the HIV status of their last male sexual partner [35]. These data indicate that people who engage in transactional sex are more prone to unwittingly spreading HIV. Forty-five percent of the participants, in this current study, had engaged in transactional sex in the past 3 months. Comparatively, only 7% of the cross-sectional study participants reported engaging in transactional sex in the past 12 months [35]. These findings suggest that SMM of color that engage in transactional sex are at greater risk for unknowingly transmitting HIV.

### 5.2. Sexual Relationship Power

Comparisons of the four latent class profiles identified how sexual relationship power influences HIV risk among SMM of color, specifically how their sexual behavior influences risk. For example, Profile 1 membership was characterized as high relationship power and was associated with safer sex practices (e.g., fewer sexual partners, lower rates of transactional sex, and drug use during sex). Profile 1 highlights a correlation between high relationship power and decreased risk of HIV related behaviors. In addition, Profile 1 members reported engaging in condom-less sex. However, members of Profile 1 had the least number of sexual partners and were much less likely to participate in other behaviors that can increase HIV transmission, such as drug use before or during sex. Although members of Profile 1 are engaging in condom-less sex, they are generally at lower risk for HIV transmission due to having fewer sexual partners and lower drugs use during sex [36,37].

Measures of relationship power in this study were obtained through self-report. However, unseen contextual internal and external factors contributed to the self-report responses. Stereotypes regarding SMM and their preferred sexual positioning also have the potential to inadvertently decrease relationship power [11,38,39]. The weight of sexual positioning in sexual relationship decision-making, for example, could be an important marker of relationship power [11,38,39]. While SMM are perceived to prefer an insertive role due to stereotypes that indicate it as the more “masculine” role [11,38,39], in reality, preferences are much more variable in sexual minority men [21,40]. Although not explicitly explored within our study, determining sexual positioning is an inevitable decision to make during a sexual encounter. Other studies have explored the many external factors, such as gender norms, that may impact sexual positioning decisions [21,39,40]. Gendered stereotypes may discourage SMM of color (especially those who engage in transactional sex) from their preferred sexual positions [21,38,39,40]. This potentially indicates a loss in sexual decision-making [11], which, as found in Profile 3 and 4, is associated with unsafe sex behaviors and higher HIV risk. Therefore, stereotypes may systematically limit the sexual roles that SMM of color can take, potentially decreasing sexual relationship power. This is one example of how societal forces may influence the intricacies of relationship power. However, the influence that stigma has on relationships may be expandable to other components of relationship power. This influence may, consequently, increase the risk of unsafe sex behaviors and, transitively, HIV transmission.

With regard to substance use on sexual relationship power, those in Profiles 2 and 4, were approximately twice as likely to use substances during sex than those in Profile 1. Substance use, especially of injection drugs, during sex increases the risk of HIV transmission [36,41,42]. Substance use during sex can affect an individual’s ability to consent and assert their sexual preferences, resulting in low sexual relationship power [22]. This, in turn, is associated with an increase in high-risk sexual behaviors, such as condom-less sex [43]. Therefore, low relationship power is associated with high-risk sexual behaviors, placing populations in Profiles 2, 3 and 4 at greater risk of HIV transmission.

These findings, in concurrence with the findings from similar studies, indicate that relationship power can be predictive of HIV transmission, within, but also outside of, sexual minority communities [10,11]. Therefore, a greater emphasis on improving sexual decision-making and sexual relationship control may have a significant effect on the transmission of HIV.

### 5.3. HIV Knowledge

High HIV knowledge may influence an individual’s sexual behaviors. Our study results indicated that membership to Profile 1 was positively associated with higher HIV knowledge, greater relationship power, decrease in and negatively associated with transactional sex, and substance use during sex. One study found an association between higher self-efficacy, use of condoms, and HIV knowledge, suggesting that HIV knowledge is associated with greater relationship power and safe sex practices [17].

In the US, Black and Latinx households are disproportionately affected by poverty, which has been associated with lower educational attainment [44]. This, in turn, may impact overall HIV knowledge [17,45]. In our sample, 1.6% of participants had less than high school education (Table 1). However, all participants who had less than a high school education were from Profiles 2 and 4. These profiles were associated with low relationship control and engagement in transactional sex. This suggests that those who have not completed high school are at a higher risk of having low HIV knowledge and, therefore, decreased relationship power. Low HIV knowledge may result in a greater risk for HIV transmission.

Our study focused on young adult men ages 18–34. Although the amount and extent of HIV prevention education via sex education varies in secondary schools, sex education has been shown to be an effective tool in increasing safe sex practices [17,46,47]. Equipping those in secondary education with high HIV knowledge may have a holistically protective effect [17]. In addition to the increased risk of HIV transmission due to decreased HIV knowledge, those who have not completed secondary education are at a higher risk of needing to rely on transactional sex for income [48]. More research is needed on interventions that are culturally tailored and appropriate for increasing HIV knowledge and increasing sexual relationship power in young adult ethnic/racial sexual minority communities.

### 5.4. Limitations

There were several limitations to this study. First, the data were cross-sectional, which does not account for causal inference or changes over time. However, we were able to assess the prevalence of HIV-risk factors represented in our latent profiles. Due to this analysis, we have identified that more interventions focusing on increasing HIV knowledge and increasing relationship power in emerging adult SMM of color are warranted. Second, desirability bias is a limitation of using self-report data. Participants could underreport responses on sensitive questions about sexual behaviors. This may have been minimized as data were collected using an online survey and not in-person, decreasing the risk of inaccurate responses. Third, although we established profiles of HIV risk that examined sexual power, transactional sex, HIV knowledge, and health literacy, using secondary data limited our analyses to variables within the dataset. Fourth, while LPA models are explicit in modeling underlaying data, there are some limitations associated with this analysis technique. For instance, LPA models assume indicators follow a specific, usually normal, distribution [49]. This can be a cause for concern when recoding continuous indicators into categorical variables [50]. Careful consideration should be given when recoding to ensure that recoded categorical indicators from continuous variables result in meaningful categories and do no result in substantial loss of information [50]. In addition, in any LPA model, the issue of reification is of great importance. With LPA it can be easy to conclude that a set of latent classes identified in the analysis are representative of the types of individuals in the population [50]. Instead, LPA is a useful heuristic for representing heterogeneity across dimensions in the model [50]. In addition, while the sample size for the current study was adequate to examine LPA groups [51], little is still known about the exact effect of sample size on the ability to identify the set of underlying latent classes; this is an important area for future research [50]. Last, generalizability to the larger SMM community is limited due to our sample size. However, the ethnic and racial diversity of the sample, usage of LPA, and our findings from this study provide unique insights into understanding the HIV risk profiles of SMM of color.

### 5.5. Implications for HIV Prevention

Overall findings from this study suggested that having low relationship power, health literacy, HIV knowledge, and engaging in transactional sex increase HIV risk in emerging adult SMM of color. Using LPA was ideal to examine the complex interrelated relationships and how they influence HIV risk.

One implication for HIV prevention for SMM who engage in transactional sex is to reduce the stigma about transactional sex within the clinical setting. Stigma and disaffirming environments create unwelcome healthcare consequences such as decreased access to care, poorer social and health outcomes, and decline in medication adherence [52,53,54,55]; in addition, they can negatively impact the psychosocial factors that motivate HIV-prevention healthcare-seeking behavior [56,57]. Therefore, providing non-judgmental clinical environments that integrate unbiased and affirming patient/provider communication with SMM that engage in transactional sex can be a powerful means to build trust and increase awareness and uptake with biomedical HIV prevention services, such as pre-exposure prophylaxis (PrEP).

The current literature on PrEP use among emerging adult SMM of color who engage in transactional sex remains limited due to the legal and criminal consequences often associated with transactional sex in the US [58]. Nevertheless, PrEP is an effective means to decrease HIV transmission among people engaging in high-risk sexual behaviors, similar to those with membership to Profiles 2 and 4. While participants who engage in transactional sex remain ideal candidates to benefit from PrEP use, one study found that high-risk sexual minority men of color are actually less likely to be prescribed and effectively use PrEP [59]. This is disheartening since SMM of color bear the highest disproportionate incidence of HIV in the US. Without a sincere effort to increase PrEP awareness and uptake in SMM of color, HIV incidence only will continue to increase.

Multiple US-based studies have concluded that SMM perceive PrEP as an effective mode of HIV prevention [57,60,61]. However, PrEP use and adherence were significantly lower in US cities when contrasted to comparable Canadian cities [62,63,64,65]. Studies indicate that societal homophobia and the stigmatization of HIV may have a significant impact on the uptake of PrEP and, consequently, the health outcomes of those at highest risk of HIV [62,66,67,68]. However, Canada has found success in targeting homophobia and social stigma surrounding HIV by enacting official health-related policy that acknowledges LGBTQ+ legitimacy in the public sphere and engages the public with this often-marginalized community [69]. Thus, healthcare policymakers and stakeholders can further maximize effective system-wide HIV-prevention strategies by prioritizing health policy that is supportive and protective to the identities and lived experiences of sexual minority men of color.

## 6. Conclusions

The purpose of this study was to examine the risk profiles of SMM of color using latent profile analysis and to identify the distinct subgroups within the profiles who carry the highest HIV risk. Based on our findings, Profile 4, SMM with lower sexual power and HIV knowledge, and higher transactional sex rates, were at the highest risk of HIV transmission. Using LPA, we were able to categorize and uncover HIV risk within four subgroups relative to others similar in the population. Our findings suggest the critical need for increasing HIV knowledge and increasing the perceived ability to make decisions in a sexual relationship (i.e., sexual relationship power) in SMM of color who engage in transactional sex and are at high risk of HIV.

## Figures and Tables

**Table 1:** Sociodemographic characteristics between latent profile groups (*N* = 322).

	Total		Profile 1: Low Transactional Sex and High Power (*n* = 133; 14.3%)		Profile 2: Transactional Sex, High Decision-Making in Sexual Relationships, and Low Control in Sexual Relationships (*n* = 99; 30.7%)		Profile 3: Low Transactional Sex, Low Decision-Making, and Moderate Control (*n* = 43; 13.4%)		Profile 4: High Transactional Sex and Low Power (*n* = 47; 14.6%)			
	*N*	%	*N*	%	*N*	%	*N*	%	*N*	%	*Χ^2^*	*p*
Gender												
Male	322	100	133	41.3	99	30.7	43	13.4	47	14.6	NA	NA
Age (M = 26.35, SD = 4.66)											0.37	0.95
18 to 24 years	114	35.5	49	36.8	35	35.7	15	34.9	15	31.9		
25 to 34 years	207	64.5	84	63.2	63	64.3	28	65.1	32	68.1		
Race–Ethnicity												
Hispanic	157	48.8	61	45.9	49	49.5	22	51.2	25	53.2	0.93	0.81
Black	122	37.9	55	41.4	33	33.3	13	30.2	21	44.7	3.54	0.31
White	74	23	26	19.5	21	21.2	14	32.6	13	27.7	3.87	0.27
Asian Identity	69	21.4	31	23.3	21	21.2	9	20.9	8	17	0.83	0.84
American Indian/Native American Identity	17	5.3	8	6	5	5.1	2	4.7	2	4.3	0.28	0.96
Middle Eastern Identity	10	3.1	0	0	6	6.1	1	2.3	3	6.4	8.9	0.03
Employment											25.78	0.01
Employed Full-time	167	52.4	65	48.9	55	56.1	19	44.2	28	62.2		
Employed Part-time	54	16.9	17	12.8	16	16.3	8	18.6	13	28.9		
Self-employed	19	6	12	9	5	5.1	2	4.7	0	0		
Unemployed	34	10.7	15	11.3	13	13.3	3	7	3	6.7		
Student	45	14.1	24	18	9	9.2	11	25.6	1	2.2		
Education											18.74	0.41
Less than high school	5	1.6	0	0	4	4	0	0	1	2.1		
High school graduate/GED	77	23.9	36	27.1	19	19.2	11	25.6	11	23.4		
Some College	75	23.3	28	21.1	27	27.3	10	23.3	10	21.3		
2-year degree	38	11.8	14	10.5	14	14.1	5	11.6	5	10.6		
4-year degree or higher	95	29.5	35	26.3	28	28.3	14	32.6	18	38.3		
Professional Degree	28	8.7	17	12.8	6	6.1	3	7	2	4.3		
Doctorate	4	1.2	3	2.3	1	1	0	0	0	0		
Income											15.15	0.65
Less than USD 10,000	59	18.3	25	18.8	21	21.2	7	16.3	6	12.8		
USD 10,000–USD 29,999	84	26.1	34	25.6	22	22.2	17	39.5	11	23.4		
USD 30,000–USD 49,999	69	21.4	30	22.6	21	21.2	5	11.6	13	27.7		
USD 50,000–USD 69,999	44	13.7	19	14.3	14	14.1	4	9.3	7	14.9		
USD 70,000–USD 89,999	29	9	9	6.8	9	9.1	6	14	5	10.6		
USD 90,000–USD 149,000	26	8.1	9	6.8	10	10.1	4	9.3	3	6.4		
More than USD 150,000	11	3.4	7	5.3	2	2	0	0	2	4.3		
Access to HIV testing outcomes											4.24	0.05
Yes	274	85.1	117	88	86	86.9	35	81.4	36	76.6		
No	48	14.9	16	12	13	13.1	8	18.6	11	23.4		

Note. Percentages may not equal 100 due to missing data.

**Table 2:** Correlation matrix among main analytic variables (*N* = 322).

	1	2	3	4	5	6	7	8
1.Power in Sexual Relationship: Control	1	0.15 **	−0.19 **	0.10 *	0.17 *	−0.08	−0.17 **	−0.35 **
2.Power in Sexual Relationship: Decision-Making		1	−0.15 *	0.10 *	−0.08	−0.03	−0.10 *	−0.01
3.Transactional Sex ^a^			1	0.07	−0.10 *	0.21 **	−0.06	0.27 **
4.Access to HIV Testing Outcomes ^a^				1	0.01	0.12 **	0.01	0.15 **
5.HIV Knowledge					1	−0.06	−0.01	−0.11 *
6.Number of Sexual Partners in Past 6 Months						1	−0.08	−0.09
7.Safe Sex Behavior: No Condom Use During Sex							1	0.22 *
8.Safe Sex Behavior: Drug Use During Sex								1

Note. ^a^ Reference Group is “no”, * *p* < 0.05, ** *p* < 0.01 (two-tailed test).

**Table 3:** Comparison of fit statistics for latent profile analysis models conducted among MSM (*N* = 322).

	LL	BIC(LL)	AIC(LL)	Npar	L^2^	df	*p*-Value	Class.Err.
1-Cluster	−978.80	1998.02	1971.60	7	70.71	24	0.00	0.00
2-Cluster	−956.79	1977.10	1935.58	11	26.69	20	0.14	0.07
3-Cluster	−951.93	1990.47	1933.86	15	16.97	16	0.39	0.18
**4-Cluster**	**−948.60**	**2006.93**	**1935.21**	**19**	**10.32**	**12**	**0.59**	**0.19**
5-Cluster	−947.98	2028.79	1941.97	23	9.08	8	0.33	0.26
6-Cluster	−946.58	2049.07	1947.16	27	6.27	4	0.18	0.32

Note. LL: Log-likelihood; AIC: Akaike information criterion; BIC: Bayesian information criterion; L^2^: likelihood ratio chi-squared statistics; df: Degrees of freedom; Class.Err: Classification Error.

**Table 4:** Mean-level group differences between latent profile groups (*N* = 322).

	Profile 1: Low Transactional Sex and High Power (*n* = 133; 14.3%)		Profile 2: Transactional Sex, High Decision-Making in Sexual Relationships, and Low Control in Sexual Relationships (*n* = 99; 30.7%)		Profile 3: Low Transactional Sex, Low Decision-Making, and Moderate Control (*n* = 43; 13.4%)		Profile 4: High Transactional Sex and Low Power (*n* = 47; 14.6%)		Total			
	*M*	*SD*	*M*	*SD*	*M*	*SD*	*M*	*SD*	*M*	*SD*	F	*p*-Value
HIV knowledge	13.44	4.39	11.44	5.05	12.44	4.87	10.9	5.06	11.51	4.76	1.12	0.05
Number of sexual partners in past 6 months	2.37	7.75	5.39	11.64	3.33	8.05	17.42	8.01	4.48	10.77	2.05	0.04
Safe sex behavior: No condom use during sex	13.49	4.47	12.53	3.65	12.79	3.62	11.8	3.36	12.85	3.99	2.44	0.04
Safe sex behavior: Drug use during sex	1.91	1.32	2.61	1.34	1.88	0.93	3.34	1.7	2.33	1.44	15.96	<0.001

**Table 5:** Multinomial logistic regression model assessing factors associated with latent profile group membership among MSM participants (*N* = 322) ^a^.

	Profile 1: Low Transactional Sex and High Power (*n* = 133; 14.3%)		Profile 3: Low Transactional Sex, Low Decision-Making, and Moderate Control (*n* = 43; 13.4%)		Profile 4: High Transactional Sex and Low Power (*n* = 47; 14.6%)	
	aOR	95% CI	aOR	95% CI	aOR	95% CI
Hispanic racial-ethnic identity ^b^	1.30	0.51, 3.33	1.36	0.47, 3.33	1.72	0.55, 5.39
White racial identity ^c^	0.45	0.15, 1.31	0.43	0.14, 1.28	0.75	0.21, 2.63
Black racial identity ^d^	1.36	0.59, 3.11	1.01	0.46, 2.63	2.43	0.86, 6.83
Employment status ^e^	1.01	0.65, 3.77	0.77	0.37, 1.58	1.29	0.56, 2.97
Access to HIV testing outcomes ^e^	2.57	1.88, 3.01	0.77	0.22, 0.99	0.38	0.13, 0.87
HIV knowledge	1.11	1.08, 1.93	1.04	1.01, 1.15	0.93	0.92, 0.99
Number of sexual partners in past 6 months	0.78	0.31, 0.99	0.49	0.08, 0.77	1.52	1.32, 3.20
Safe sex behavior: No condom use during sex	0.88	0.80, 0.98	0.92	0.81, 0.99	1.12	1.09, 1.24
Safe sex behavior: Drug use during sex	0.52	0.39, 0.68	0.64	0.33, 0.72	1.92	1.46, 2.51

Note. Bold values had *p* < 0.05. *CI =* confidence interval, *aOR* = adjusted odds ratio. ^a^ Reference level: Profile 2: Moderate Rates of Exchange for Sex and Minimal Power; ^b^ defined as “1 = yes Hispanic” versus “no = 0”; ^c^ defined as “1 = yes white” versus “no = 0” ^d^ defined as “1 = yes Black” versus “no = 0”; ^d^ defined as “1 = yes employed full-time” versus “no = 0”; ^e^ defined as “1 = yes had a HIV test” versus “no = 0”.

## Data Availability

The data presented in this study are not publicly available due to privacy and ethical considerations on protecting sexual identity and HIV status of the study participants.
